# Human organoids for Risk Group 4 virus research: a new frontier in investigating Nipah virus infection of the central nervous system

**DOI:** 10.1128/jvi.01070-25

**Published:** 2025-10-29

**Authors:** Gabriella Worwa, Shuǐqìng Yú, Amanda M. W. Hischak, Julie P. Tran, Jeremy J. Bearss, John Bernbaum, Daniel B. Woodburn, Bapi Pahar, Jillian Geiger, Louis M. Huzella, Santiago Vidal Freire, Ian Crozier, César Muñoz-Fontela, Gustavo Palacios, Nicole C. Kleinstreuer, Lina Widerspick, Jens H. Kuhn

**Affiliations:** 1 Integrated Research Facility at Fort Detrick, National Institute of Allergy and Infectious Diseases, National Institutes of Health, Frederick, Maryland, USA; 2 Department of Microbiology, Icahn School of Medicine at Mount Sinai 5925, New York, New York, USA; 3 Clinical Monitoring Research Program Directorate, Frederick National Laboratory for Cancer Research 437329 , Frederick, Maryland, USA; 4 Bernhard-Nocht-Institute for Tropical Medicine 14888, Hamburg, Germany; 5 German Center for Infection Research, Partner Site Hamburg-Lübeck-Borstel-Riems, Braunschweig, Germany; 6 Global Health Emerging Pathogens Institute, Icahn School of Medicine at Mount Sinai 5925, New York, New York, USA; 7 Division of Program Coordination, Planning, and Strategic Initiatives, National Institutes of Health 2511, Bethesda, Maryland, USA; University Medical Center Freiburg, Freiburg, Germany

**Keywords:** central nervous system infection, complex *in vitro* system, human cerebral organoid, maximum containment, NAMs, new approach methodologies, Nipah virus, NiV Bangladesh, NiV Malaysia, replication kinetics

## Abstract

New approach methodologies, such as high-complexity *in vitro* systems, are increasingly prioritized in biomedical research as potential alternatives to animal experimentation. We show that cerebral organoids derived from human induced pluripotent stem cells can be leveraged to (i) investigate isolate-specific replication dynamics of Nipah virus and (ii) model key histopathological lesions found in the brain tissue of infected human patients. Furthermore, we discuss the importance of organoid models for the study of Risk Group 4 viruses.

**IMPORTANCE:**

Advanced development of medical countermeasures against Risk Group 4 viruses, such as the Nipah virus, historically required testing in mammals under the FDA Animal Rule and translation of data to inform clinical trials in humans. Because the application of human organoids in research on viruses pathogenic for humans is conspecific, it bears the potential to reduce, refine, or replace animal studies where unnecessary. Human cerebral organoids are three-dimensional cell aggregates that resemble the developing human brain functionally and structurally. Brain organoids may be valuable in investigating the replication, neuroinvasion, pathogenesis, virulence, and persistence of neurotropic viruses and provide scientific discernment when developing medical countermeasures destined for the human end-user.

## INTRODUCTION

Nipah virus (NiV), a zoonotic and neurotropic henipavirus in the mononegaviral family *Paramyxoviridae*, is endemic to regions in Southeastern Asia, in particular Bangladesh, India, Malaysia, and the Philippines. Flying foxes of the *Pteropus* genus host NiV naturally and occasionally, during active shedding pulses, transmit the virus to other mammals, including humans, via bat excreta-contaminated food sources, contact with non-food fomites, or inhalation of aerosols (1–3).

In humans, a hallmark of central nervous system (CNS) infection is vasculitis and endothelial cell necrosis (4), but NiV readily infects cells of the CNS parenchyma, including neurons and microglia (5). CNS involvement in NiV encephalitis frequently causes headaches, altered consciousness, and death (6, 7). Survivors may experience neurological sequelae, including headaches, seizures, static encephalopathy, oculomotor dysfunction, cognitive dysfunction, bradykinesia, cervical dystonia, and facial paralysis, and may even suffer from relapsing encephalitis (6, 7).

Significant differences in the transmission, clinical disease phenotypes, and lethality/case-fatality rates of Bangladeshi (NiV-B) and Malaysian (NiV-M) isolates have been described in both natural and experimental infections. Compared with NiV-M, NiV-B appears to transmit more efficiently (thereby contributing to increased person-to-person spread) and has a shorter incubation period. Encephalitis occurs after infection with both isolates, but NiV-B infection is associated with a higher frequency of respiratory disease and higher overall case-fatality rates. Data from experimental exposures of nonhuman primates largely confirmed these differences (8, 9). When grivets were exposed intranasally and intratracheally, NiV-B caused more severe clinical signs than NiV-M, resulting in 100% lethality, whereas NiV-M resulted in 50% lethality in this study and another that tested only intratracheal exposure (9). However, as with most nonhuman primate studies, these data were derived from small group sizes (*n* = 3 or 4), and the publications do not specify whether study personnel were blinded.

The host-virus exposure determinants of these between-isolate differences in disease severity and phenotype are understudied, including in the CNS. Indeed, “side-by-side” comparisons of cellular tropism, viral replication, and cellular/tissue damage have not been possible in human, animal model, or 2D *in vitro* systems.

Here, we describe the first effort to model these characteristics of NiV infection using a new approach methodology (NAM), that is, human cell-based organoids as a potential future alternative to animal experimentation.

## RESULTS

### Distinct Nipah virus isolates replicate with distinct kinetics in human cerebral organoids

NiV is a neurotropic virus. In a human biology-based *in vitro* system, we sought to determine whether NiV replication and neurovirulence, including after exposure to the two major isolates, NiV-B and NiV-M, can be investigated in human cerebral organoids. In a first proof-of-concept experiment, we differentiated human induced pluripotent stem cells (iPSCs) and subsequently matured matrix-embedded cerebral organoids for a total of 3 mo. All personnel examining endpoints as part of this experiment remained blinded to the type of exposure until the conclusion of the experiment and subsequent data analysis.

**Fig 1:**
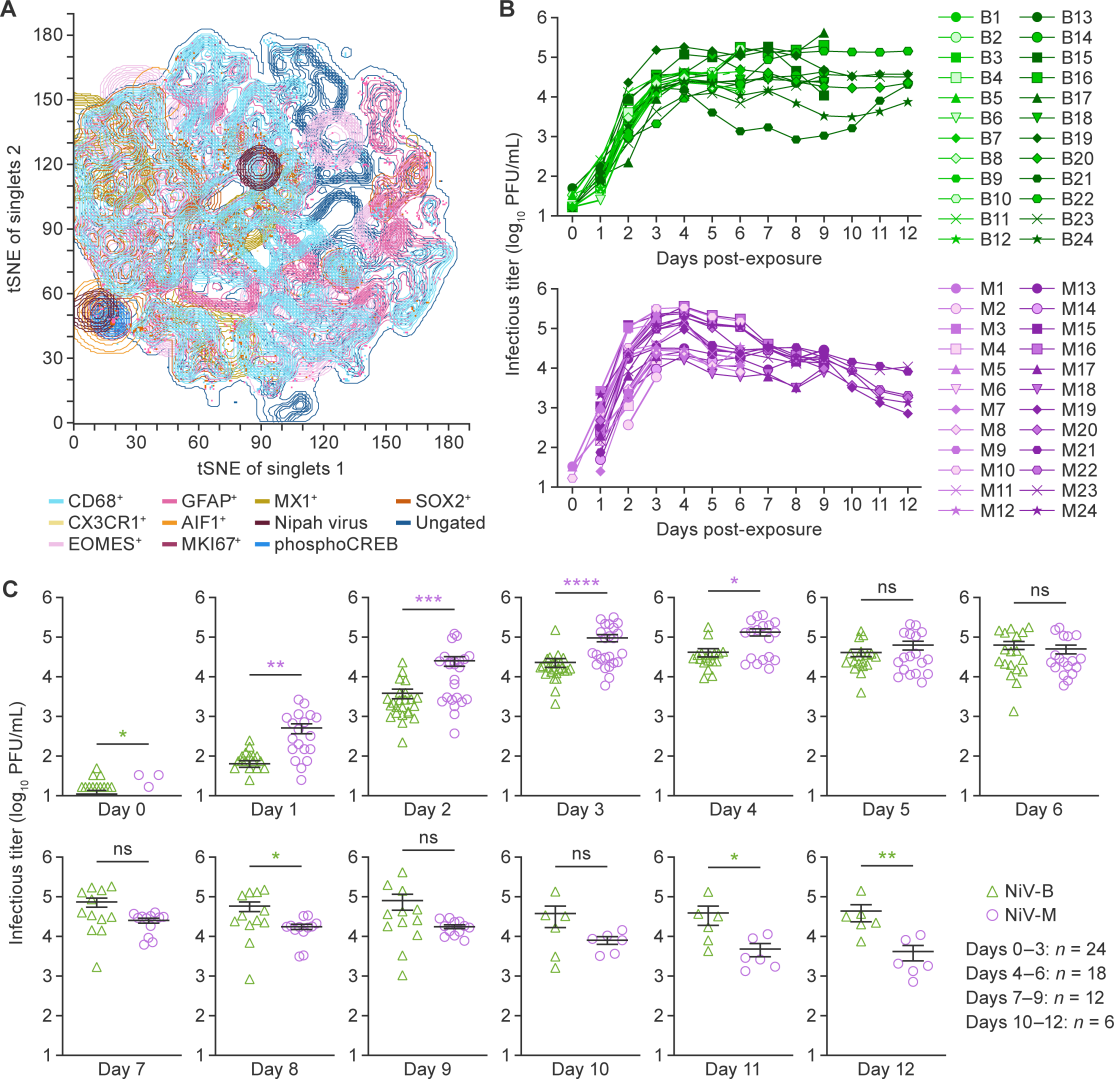
Distinct Nipah virus (NiV) isolates replicate with distinct kinetics in human cerebral organoids. (**A**) *t*-distributed stochastic neighbor embedding (*t*-SNE) plot showing the different subsets of cells positive for glial fibrillary acidic protein (GFAP^+^) in a cerebral organoid not exposed to NiV (to discern features, zooming into the figure of the electronic version of this article is recommended). Staining was performed with antibodies for macrophages (CD68 molecule [CD68]), astrocytes (GFAP), stem cells (SRY-box transcription factor 2 [SOX2]), neuronal differentiation factors (Ser133-phosphorylated cAMP responsive element binding protein [phosphoCREB]), virus (NiV, in-house), MX dynamin like GTPase 1 (MX1), marker of proliferation (Ki-67 [MKI67]), microglial cells (allograft inflammatory factor 1 [AIF1]), eomesodermin (EOMES), and a chemokine receptor (C-X3-C motif chemokine receptor 1 [CX3CR1]). (**B**) On Day 0, a total of 72 cerebral organoids were exposed to either NiV Bangladesh (NiV-B), NiV Malaysia (NiV-M), or mock/media (*n* = 24 per group). Organoid culture media were collected daily, and viral titers were determined by plaque assay titration for a total of 12 d post-exposure. Because six organoids per ger group were collected on Days 3, 6, 9, and 12 post-exposure, the group sizes decreased as the experiment progressed and was as follows: *n* = 24 at 0–3 d, *n* = 18 at 4–6 d, *n* = 12 at 7–9 d, and *n* = 6 at 10–12 d. Organoids for which no PFUs were detected (infectious titer = 0 PFU/mL, technically “undefined” on a log_10_ scale) are not shown, as in Day 0. NiV-B is shown in green (B1–B24); NiV-M is shown in purple (M1–M24); mock/media is not shown. (**C**) A Mann-Whitney test was performed to assess the significance of differences in titers between NiV-B and NiV-M for each day. *P* values were calculated based on median titers from the indicated numbers of organoids/group. *P* ≥ 0.05 = not significant (ns); *P* ≤ 0.05 (*); *P* ≤ 0.01 (**); *P* ≤ 0.001 (***); *P* ≤ 0.0001 (****).

As proof of principle that spatial clustering and size of cell populations present in the organoids can be visualized, we stained an unexposed organoid with specific antibodies targeting astrocytes (cells positive for glial fibrillary acidic protein [GFAP^+^]). A *t*-distributed stochastic neighbor embedding (*t*-SNE) plot revealed numerous macrophages positive for CD68 molecule (CD68^+^) and smaller areas of GFAP^+^ astrocytes with interspersed undifferentiated stem cells positive for SRY-box transcription factor 2 (SOX2^+^). An area positive for Ki-67 marker of proliferation (Ki67^+^), indicating proliferative activity, overlapped with an area positive for allograft inflammatory factor 1 (AIF1^+^) microglial cells and signs of neuronal differentiation as judged by detection of Ser133-phosphorylated cAMP responsive element binding protein (phosphoCREB^+^) (Fig. 1A).

Next, we transferred cerebral organoids into maximum (biosafety level 4) containment for exposure to 10,000 PFU of NiV-B, NiV-M, or mock/media on Day 0. Organoid culture media were collected immediately after removal of inocula, and every 24 h until Day 12 post-exposure to generate growth curves. Plaque assays were performed using these media to quantify the infectious titers and define replication kinetics of both NiV isolates in the organoids over time (Fig. 1B). A small, but significantly higher NiV-B titer difference was noted (*P* = 0.01; *n* = 24) as residue following inoculation compared with NiV-M on Day 0, but we left titers unadjusted to track replication kinetics neatly. We observed an increase in NiV-B titers, which remained elevated until Day 4 to 3.96–5.26 log_10_ PFU/mL, followed by a plateau, except for organoids #B22 and #B24 (Fig. 1B). In contrast, we observed a distinct peak of NiV-M replication on Day 4, with titers ranging between 4.20 and 5.56 log_10_ PFU/mL, followed by a steady decline in titers until Day 12 (Fig. 1B). The final titers of organoids (*n* = 6 per group) remaining on Day 12 were 3.87–5.15 and 2.86–4.04 log_10_ PFU/mL for NiV-B and NiV-M, respectively (Fig. 1B). Despite the small increase in titers of NiV-B after inoculation (Day 0), NiV-M replicated to significantly higher titers at 1-4 d (Fig. 1C); titers were similar during subsequent time points (at 5–7 d) and, ultimately, higher for NiV-B at 11 and 12 d in accordance with the replication plateau described above (Fig. 1B and C). Overall, we observed slightly different replication dynamics in cerebral organoids: NiV-M replicated more efficiently early to a peak and subsequent small decline, whereas NiV-B replicated more slowly to a prolonged plateau over the course of the experiment.

Overall, we show that cerebral organoids usefully enable side-by-side investigation of viral replication dynamics, thus far not possible in human, animal model, or 2D tissue culture systems (8–11).

### Distinct Nipah virus isolates cause characteristic histopathological lesions in human cerebral organoids

**Fig 2:**
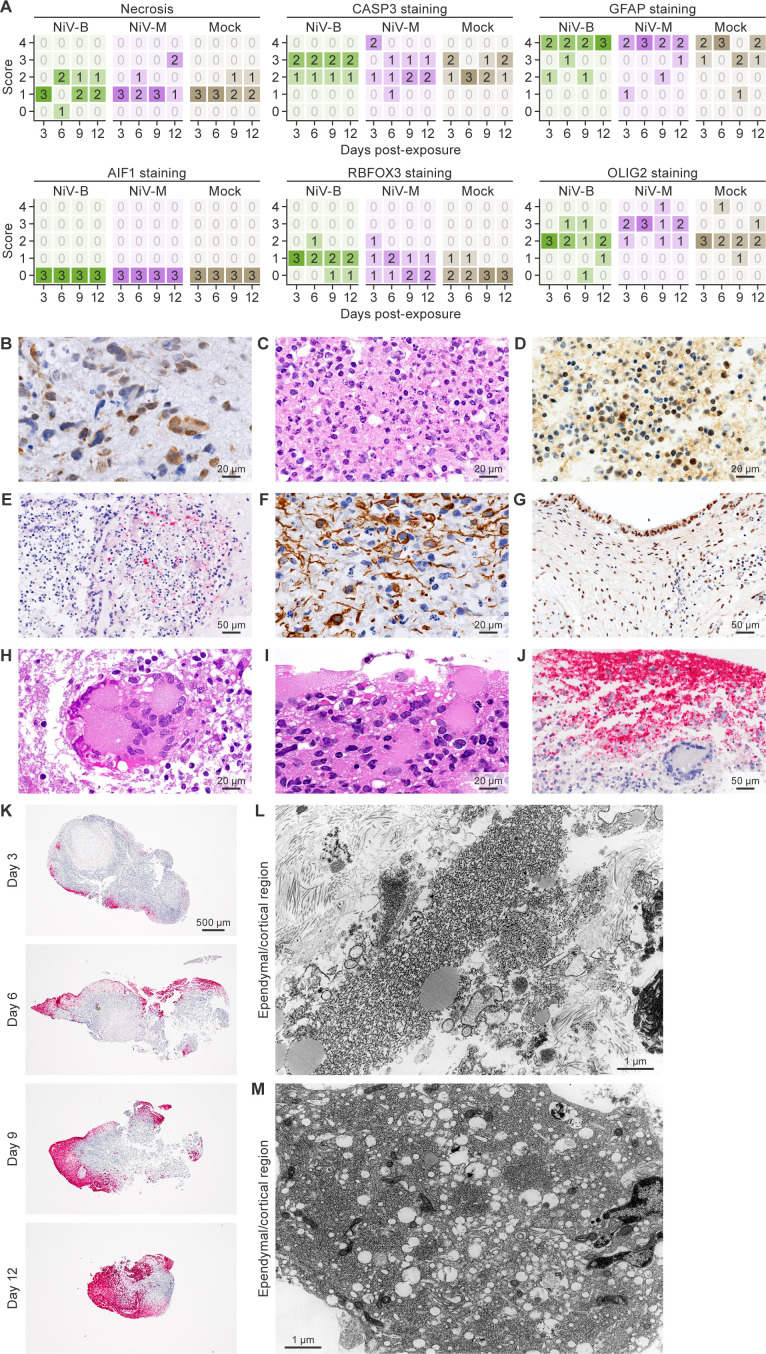
Distinct Nipah virus (NiV) isolates cause characteristic histopathological lesions in human cerebral organoids. At 3, 6, 9, and 12 d post-exposure, three cerebral organoids per group were microscopically assessed: one half of each organoid was used for various histopathologic stains, followed by light/immunofluorescence microscopy; the other half was examined by transmission electron microscopy (TEM). (**A**) All three organoids per group/day were assigned scores of 0–4 to aid in grading the presence and extent of necrosis (caspase 3 [CASP3] staining) and expression of astrocyte marker glial fibrillary acidic protein (GFAP), microglial marker allograft inflammatory factor 1 (AIF1), nuclear protein marker of mature neurons (RNA binding fox-1 homolog 3 [RBFOX3]), and oligodendrocyte marker (oligodendrocyte transcription factor 2 [OLIG2]). Scores: 0 = not detected; 1 = 1%–10%; 2 = 11%–30%; 3 = 31%–50%; 4 = >50%. NiV Bangladesh (NiV-B) is shown in green; NiV Malaysia (NiV-M) is shown in purple; and mock is shown in gray. Increasing color intensity corresponds to increasing numbers of organoids assigned the respective score. (**B**) Representative immunohistochemical (IHC) staining of a NiV-B-exposed cerebral organoid on Day 6 for RBFOX3 (magnification: 1,000×). (**C**) Representative hematoxylin and eosin (H&E) staining of a NiV-M-exposed cerebral organoid on Day 6 showing widespread necrosis (magnification: 1,000×). (**D**) Representative IHC staining of a NiV-M-exposed cerebral organoid on Day 6 for CASP3, revealing widespread cell death (magnification: 1,000×). (**E**) Representative IHC staining of a mock-exposed cerebral organoid on Day 6 for AIF1 (magnification: 400×). (**F**) Representative IHC staining of a mock-exposed cerebral organoid on Day 3 for GFAP (magnification: 1,000×). (**G**) Representative IHC stain of a NiV-B-exposed cerebral organoid on Day 6 for OLIG2 (magnification: 400×). (**H**) Representative H&E stain of a NiV-B-exposed cerebral organoid on Day 9, uncovering NiV-typical syncytia formation (magnification: 1,000×). (**I**) Representative H&E stain of a NiV-M-exposed cerebral organoid on Day 6, highlighting nuclear and cytoplasmic inclusions (magnification: 1,000×). (**J**) Representative *in situ* hybridization (ISH) stain of a NiV-B-exposed cerebral organoid on Day 6 showing NiV-B genomic nucleic acids (magnification: 400×). (**K**) Series of cerebral organoids exposed to NiV-B. Halves were stained for NiV genomic nucleic acid showing progressive/spreading infection over time at 3, 6, 9, and 12 d (magnification: 40×). (**L, M**) TEM images of NiV-M-exposed and NiV-B-exposed cerebral organoids on Day 6 and Day 12, respectively, showing NiV-typical syncytia and nucleocapsid inclusions (magnification: 2,900×).

At 3, 6, 9, and 12 d post-exposure, three cerebral organoids per group were collected and bisected: one half was fixed in 10% neutral buffered formalin and assessed by histopathologic examination with immunohistochemical staining (by a board-certified veterinary pathologist who was blinded to the type of exposure) and the other half was fixed in 2.5% glutaraldehyde and 2.0% paraformaldehyde and assessed by transmission electron microscopy (TEM). All organoids examined by both light and electron microscopy had clearly identifiable cell types and a similar structural morphology that consistently included three characteristic regions. The outer, ependymal layer was thin and consisted of one to three ependymal cell layers. Beneath the ependymal layer(s), a cortical (“cortex”) layer, consisting of neurons, astrocytes, microglia, and oligodendrocytes, was reliably present but varied in thickness, ranging from six to 20 cell layers among organoids. In all organoids, the cortical layer surrounded a large inner core region consisting of various cell types and materials, including fibroblasts, collagen, matrix proteins, and areas with lysed cells. Because we found consistent morphology among all organoids, we used only one batch of organoids for this experiment. After exposure to NiV, organoid morphology was altered as progressive cell lysis increasingly led to disruption of the structural, “multi-layered” organization of the organoid. The degree of staining for GFAP^+^ and AIF1^+^, markers of necrosis (cleaved caspase 3 [CASP3]), mature neurons (RNA binding fox-1 homolog 3 [RBFOX3]), and oligodendrocytes (oligodendrocyte transcription factor 2 [OLIG2]), was graded on a scale of 0–4 for each organoid and found to be similar between virus-exposed groups (Fig. 2A). Necrosis (a common finding, especially in the organoid centers) was present in all organoids to comparable degrees (Fig. 2A). Representative images for each stain are shown in Fig. 2B through G. Cytopathic effects characteristic of NiV infection, including syncytia, nuclear and cytoplasmic viral inclusions, and nuclear atypia, were frequently observed in ependymal cells and cortical cells in all NiV-exposed organoids (Fig. 2H and I). We observed a progressive loss of ependymal cells and connective tissue in all NiV-exposed organoids but not in mock-exposed organoids. Unsurprisingly, NiV genomic nucleic acids were detected by *in situ* hybridization (ISH) in the outer/cortical regions of organoids at early time points; subsequently, increasing ISH signal with outer-to-inner progression was detected over the course of 3, 6, 9, and 12 d post-exposure (Fig. 2J and K). TEM images of NiV-exposed organoids revealed large quantities of NiV nucleocapsid inclusions (present in several cell types), widespread areas of syncytia, fused, or necrotic cells (Fig. 2L and M), and budding of mature NiV particles.

Together, these data demonstrate that NiV infection of cerebral organoids results in key pathological features, including syncytia formation and viral cytopathic effects noted in brain autopsies of humans who died with NiV encephalitis (2, 12).

## DISCUSSION

Recognized as a Biodefense Pathogen by the U.S. National Institute for Allergy and Infectious Diseases (13) and other organizations (14), NiV is a priority focus of current research and medical countermeasure (MCM) development efforts globally. In the past, researchers approached candidate MCM assessment for Risk Group 4 pathogens such as NiV via the U.S. Food and Drug Administration (FDA) “Animal Rule,” that is, the replacement of human clinical trials that cannot be performed due to ethical reasons with “well-characterized” animal models (15). Recent developments in organoid and other microphysiological system approaches, that is, NAMs may complement or possibly replace MCM evaluation in animal models. Indeed, the FDA recently announced its intention to strategically phase out reliance on the use of animal models for preclinical safety assessment of a broad range of therapeutics (16). Although the traditional use of nonhuman primates, for instance, served as a translational proxy for humans, the application of human-origin, three-dimensional organoids that mimic the anatomy and function of organs is attractive as a potential future substitute because such organoids eliminate species-related and therefore immune system-related variability, especially in the context of viral infection and MCM evaluation. Organoid creation based on the inclusion of stem cells from donors with varied and representative genetic backgrounds additionally enables organoid customization and forward-thinking diversification of experiments, thereby bringing precision medicine to the doorstep of Risk Group 4 pathogen research. Together with the ability to design blinded experiments that include high numbers of replicates (compared with the historically very low replicates of typically unblinded nonhuman primate studies), data sets derived from organoids and other NAMs provide the opportunity to robustly build confidence in experimental outcomes.

With NAMs as a focus in the FDA’s “Modernization Act 2.0” (17) and the recently announced commitment by the National Institutes of Health to prioritize human-based technologies for biomedical research (18), it is expected that maximum (biosafety level 4) containment laboratories will start to pivot towards incorporating NAMs into their research portfolios. The application and handling of organoids within maximum containment are relatively straightforward: no specialized equipment or incubators are required; furthermore, because organoid tissue resembles animal tissue, organoids can easily be incorporated into established sample/pathogen inactivation protocols and Select Agent removal workflows for downstream analyses outside of containment.

Overall, we provide proof of principle that cerebral organoids can be used in the maximum containment setting to investigate NiV viral replication and characterize infection-associated tissue damage. To the best of our knowledge, side-by-side comparisons of NiV-B and NiV-M replication or tissue damage, that is, neurovirulence, in relevant CNS cell types have not been reported in humans, animal models, or 2D cell cultures.

We acknowledge the limitations of this first effort that reflect the considerable challenges associated with the use of any new technology. Ideally, experiments in the future should include at least two “batches” of organoids from the same iPSC donor (to account for potential batch-to-batch variability) as well as from multiple donors (e.g., that might vary in age or sex), depending on the goals of the experiment. Although progress has been made, optimization of histopathologic and immunologic assays for use in virus-exposed CNS organoid systems is needed (19). For instance, we demonstrated via a *t*-SNE plot that spatial clustering and size of cell populations can be visualized in a single organoid (Fig. 1A). Because this plot represented only the cells contained in one organoid (and cannot represent the heterogeneity likely present between organoids of the same batch), we intentionally did not provide quantitative characterization, which would of course be of future interest to characterize both unexposed and NiV-exposed organoids. We hope that this work (including our staining panel and methodology) will facilitate such analyses in the future. In addition, it will be of interest to characterize cytokine secretion from individual organoids, determine whether these data can be extrapolated to all organoids of a batch (given their morphological variabilities), and then compare those global cytokine responses to those known from animal model experimentation. Also, natural NiV infection causes endothelial cell necrosis, widespread microinfarction, and vasculitis-induced thrombosis (4)—features that are necessarily absent in the cerebral organoids used for this study, given the absence of blood vessels. These features may be better mimicked in microfluidic systems (“organs-on-chips”) such as a brain-on-chip that houses microvascular endothelial cells and forms a blood–brain barrier. In organ-on-chips, experimental conditions (such as virus infection, therapeutic dosing, or addition of immune cells) can be controlled more precisely compared with organoids due to the existence of fluidic channels (including a vascular component) and a physically adaptable environment. The combination of NAMs will likely result in complementary and possibly synergistic data that may make the replacement of animal experimentation with more human-relevant models an achievable reality.

## MATERIALS AND METHODS

### Cerebral organoid generation

Human induced pluripotent stem cells (Healthy Control Human iPSC Line, Female, SCTi003-A, #200-0511, Stemcell Technologies, Cambridge, MA, USA) were used for the establishment and maturation of cerebral organoids following a commercial protocol using a STEMdiff Cerebral Organoid Kit (Stemcell Technologies). Briefly, for organoid formation (which took place from Day −10 to Day 5), one vial of frozen human iPSC was thawed on Day −10, and the cells were seeded into one well of a Matrigel-coated 6-well plate containing iPSC seeding medium. Cells were passaged three times prior to induction. On Day 0 (organoid embryoid body induction), high-quality iPSCs (distinct colonies, sharp edges, >60%) were harvested, counted, and seeded (9,000 cells per well) in a round-bottom, ultra-low-attachment 96-well plate containing organoid formation medium. On Days 2 and 4 post-seeding, 100 µL of fresh organoid formation medium were added. Prior to neuronal induction (5–7 d), the presence of organoid embryoid bodies with round and smooth edges was confirmed by light microscopy. On Day 5, the seeding/formation medium was removed and 250 µL of induction medium were added per well, and the plate was incubated for 48 h at 37°C and 5% carbon dioxide (CO_2_). For organoid expansion (7–10 d), each organoid embryoid body was drawn up in 25 µL of medium using a wide-bore pipette tip and transferred to a mold on the embedding surface. Excess medium was removed from each mold, and 15 µL of Matrigel were added dropwise onto each embryoid body. Matrigel-embedded embryoid bodies were placed in a container and incubated at 37°C and 5% CO_2_ for 30 min to achieve polymerization. Next, the embedding surface containing the Matrigel droplets was grasped, and expansion medium was used to carefully wash off each Matrigel droplet from the embedding surface into a well of an ultra-low-attachment 6-well plate. Embedded organoids were subsequently incubated in 3 mL of expansion medium at 37°C and 5% CO_2_ for 3 d. Budding of the organoids’ surfaces was monitored under the light microscope to document the development of neuroepithelia. On Day 11, wide-bore pipette tips were used to transfer expanded organoids to ultra-low-attachment 24-well plates containing 1 mL of maturation medium per well (one organoid per well to avoid fusion of multiple organoids). For organoid maturation (11–89 d), the medium was replaced with 1 mL of maturation medium per well without disturbing the embedded organoids. Plates containing organoids were then transferred onto an orbital shaker at 37°C for incubation and maturation. Maturation medium was exchanged every 3–4 d and growth was monitored weekly using light microscopy until virus exposure on Day 89. Prior to virus exposure, cerebral organoids were randomly assigned to experimental groups.

### Antibody staining

A single-cell suspension of each cerebral organoid was achieved using the Papain Dissociation System protocol (#LLK003150, Worthington Biochemical Corp, Lakewood, NJ, USA). Whole-organoid staining was performed using the following antibodies: Alexa Fluor 594 Anti-GFAP Antibody (Clone 2E1.E9, #644708, BioLegend, San Diego, CA, USA), Brilliant Violet 785 Anti-Human CD68 Antibody (Clone Y1/82A, #333826, BioLegend), PerCP/Cyanine5.5 Anti-Human CX3CR1 Antibody (Clone 2A9-1, #341614, BioLegend), Phospho-CREB (Ser133) (4D11) Rabbit mAb APC Conjugate (Clone CREBS133-4D11, #2129, AbwizBio, San Diego, CA, USA), BD Horizon BV605 Mouse Anti-Ki-67 (Clone B56, #567122, BD Biosciences, Franklin Lakes, NJ, USA), BD Horizon BUV395 Mouse Anti-EOMES (Clone X4-83, #567171, BD Biosciences), Pacific Blue Anti-SOX2 Antibody (Clone 14A6A34, #656112, BioLegend), Alexa Fluor 750 MxA/Mx1 Antibody OTI2G12 (Clone OTI2G12, #NBP2-72838AF750, Novus Biologicals), and anti-NiV antibody (in-house). The AIF-1/Iba1 Antibody, goat, polyclonal (#NB100-2833, Novus Biologicals, Centennial, CO, USA) was used in an in-house custom conjugation process utilizing a PE-Cy7 Conjugation Kit (#ab102903, Abcam, Cambridge, UK). Live/dead cells were identified with a Live/Dead Fixable Aqua Dead Cell Stain Kit, at 405 nm excitation (#L34966, Thermo Fisher Scientific, Waltham, MA, USA).

Initially, the organoid samples were stained for surface markers. Subsequently, the cells were permeabilized using Cytofix/Cytoperm (# 554714, BD Biosciences), followed by intracellular staining. The cells were subsequently washed and fixed with 500 µL of Cytofix/Cytoperm, resuspended in phosphate-buffered saline (PBS), and data acquisition was performed using a five-laser Cytek Aurora Flow Cytometer (Cytek Biosciences, Fremont, CA, USA). The *t*-SNE analysis was performed using FlowJo software version 10.8.1.

### Virus exposure

On Day 0, 89-day-old cerebral organoids were exposed in maximum (biosafety level 4) containment to either mock/media or 10,000 PFU of a laboratory stock of NiV (*Paramyxoviridae: Henipavirus nipahense*), isolate Bangladesh 2004 (GenBank accession no. PV892943), or isolate Malaysia 1998 (GenBank accession no. PQ463988) diluted in 500 µL of STEMdiff Cerebral Organoid Basal Medium with Supplement E (#08571, Stemcell Technologies; henceforth: organoid media). After incubation for 1 h at 37°C, inocula were removed, and organoids were washed once in 1 mL of Gibco Dulbecco’s PBS (DPBS; #14190094, Thermo Fisher Scientific), followed by the addition of 1.5 mL of fresh organoid media. Daily harvests of organoid media were used for plaque assay titration as described previously (20).

### Histopathology

Cerebral organoids were carefully bisected, and halves were fixed in either 10% neutral-buffered formalin (for histology) or in 2.5% glutaraldehyde/2.0% paraformaldehyde (for transmission electron microscopy) for at least 72  h prior to removal from maximum containment.

Formalin-fixed organoid halves were routinely processed with an optimized abbreviated protocol in a Tissue-Tek VIP-6 vacuum infiltration automated tissue processor (Sakura Finetek USA, Torrance, CA, USA), paraffin-embedded using a Tissue-Tek TEC-6 embedding console system (Sakura Finetek), sectioned at 4  µm using a standard semiautomated rotary microtome (Leica RM2255, Leica Biosystems), mounted on positively charged uncoated glass slides, and then air-dried for routine hematoxylin and eosin (H&E) staining.

Organoid slides were immunohistochemically stained for rabbit anti-GFAP (#Z0334, Dako, 1:3,000 dilution), Anti-NeuN antibody EPR12763 Neuronal Marker (RBFOX3, #ab177487, Abcam, 1:3,000 dilution), Anti-OLIG2 Antibody (#AB9610, Millipore, 1:100 dilution), Anti-Iba1 antibody (AIF1, #ab107159, Abcam, 1:1,300 dilution), and Cleaved Caspase-3 (CASP3) Antibody 9661 (#9661L, Cell Signaling, 1:150 dilution). All tissues were visualized with either 3,3'-diaminobenzidine brown chromogen (#BDB2004L, Betazoid DAB Chromogen Kit, Biocare Medical) or red chromogen (#WR806S, Warp Red Chromogen Kit, Biocare Medical) hematoxylin (blue) counterstain. Sections were examined by light microscopy and scored with a subjective percentage of positive cells.

RNAscope *in situ* hybridization was performed to detect NiV genomic nucleic acids in sections of each organoid. Routinely processed 4 µm formalin-fixed paraffin-embedded tissue sections were mounted on positively charged uncoated glass slides and stained using a manual RNAscope 2.5 HD Red kit (#322360, Advanced Cell Diagnostics, Newark, CA, USA), following the manufacturer’s protocol, including modifications for optimization validated by appropriate controls. A synthesized 20-ZZ pair probe targeting the NiV nucleoprotein (*N*) gene was used (V-Nipah-StrM.B.-N, #439251, Advanced Cell Diagnostics), and the sections were counterstained with hematoxylin and glass-coverslipped with a non-alcohol-based mounting medium to prevent chromogen fading. Sections were examined by light microscopy and scored with a subjective percentage of positive cells.

### Transmission electron microscopy

For conventional thin-section microscopic evaluation, organoids were preserved and inactivated in 2.5% glutaraldehyde/2.0% paraformaldehyde (Electron Microscopy Sciences, Hatfield, PA, USA) in Millonig’s Sodium Phosphate Buffer (Tousimis Research, Rockville, MD, USA). After 72 h of fixation, organoids were washed in the same buffer and incubated for 2 h in 1.0% osmium tetroxide (Electron Microscopy Sciences). After rinsing in water and *en bloc* staining with 2.0% uranyl acetate (Ted Pella, Redding, CA, USA), samples were dehydrated in a series of graded ethanols and then infiltrated and embedded using a SPURR Low-Viscosity Embedding Kit (Electron Microscopy Sciences). After polymerization, embedded blocks were cut into 70–80-nm sections using an EM UC7 ultramicrotome (Leica Microsystems, Deerfield, IL, USA). Sections were collected on 150-mesh copper grids, stained with lead citrate, and examined using a FEI Tecnai Spirit twin-transmission electron microscope (model G2 F20, Thermo Fisher Scientific) operating at 80 kV.

## Data Availability

All data are contained within this article.
